# Reduced half-life of recombinant ADAMTS13 in a patient with cTTP undergoing total hip arthroplasty

**DOI:** 10.1186/s12959-025-00792-y

**Published:** 2025-10-27

**Authors:** Maria Weise, Thomas Siegemund, Tristan Klöter, Maren Keller, Sirak Petros, Christian Pfrepper

**Affiliations:** 1 Division of Hemostaseology, Department of Hematology, Cellular Therapy and Hemostaseology, University of Leipzig Medical Center, Leipzig, Germany; 2 MVZ Limbach, Magdeburg, Germany; 3 Division of Anesthesiology and Intensive Care, University of Leipzig Medical Center, Leipzig, Germany; 4 Medical ICU, University of Leipzig Medical Center, Leipzig, Germany

**Keywords:** Congenital thrombotic thrombocytopenic purpura, Recombinant ADAMTS13, Surgery

## Abstract

A 41 year old female with congenital thrombotic thrombocytopenic purpura (cTTP) underwent a total hip arthroplasty under prophylaxis with recombinant ADAMTS13 (a disintegrin and metalloproteinase with thrombospondin motifs 13). The procedure was performed without complications 12 h after the last injection. Postoperative ADAMTS13 activity levels showed a more rapid decrease in activity and in the individual apparent half-life due to increased consumption of the recombinant protease that could safely be managed by an intensified dosing regimen.

## Introduction

Congenital thrombotic thrombocytopenic purpura is an ultra-rare disease with an estimated prevalence of 0.5–2.5 per million with incidence peaks in the neonatal phase and early adulthood [1]. cTTP is caused by homozygous or compound heterozygous mutations in the ADAMTS13 gene that lead to a severe deficiency in ADAMTS13 with an activity < 10% compared to normal plasma [2]. The deficiency causes microvascular thrombosis with end-organ damage, thrombocytopenia and microangiopathic hemolytic anemia due to persisting ultra-large von-Willebrand multimers binding and activating platelets.

Severe ADAMTS13 deficiency alone is usually not sufficient to develop an acute episode of cTTP. Conditions such as pregnancy, infections, excessive alcohol consumption and surgery can result in an acute exacerbation [3, 4]. Pathophysiological, this is explained by increased von Willebrand factor levels in plasma, endothelial cell activation, and increased shear stress [5].

For many years infusions of fresh frozen plasma (FFP) were administered for treatment of acute and prophylaxis of recurrent episodes in cTTP [6]. However, the recent first phase-3-trial has demonstrated the safety and efficacy of recombinant ADAMTS13 (rADAMTS13) in comparison to routine and on-demand therapy with plasma infusions [7]. Side effects of rADAMTS13 were low, no neutralizing antibodies against rADAMTS13 have been detected so far and none of the patients on prophylaxis with rADAMTS13 developed an acute TTP. The median half-life of rADAMTS13 is 46.6 h (range 28.4 to 83.3) [7]. United States Food and Drug Administration (09/2023), Pharmaceuticals and Medical Devices Agency in Japan (03/2024) and European Medicines Agency (08/2024) approved rADAMTS13 (ADAMTS13 recombinant-krhn, Adzynma^®^, Takeda Manufacturing Austria AG) for treatment of cTTP in children and adults.

## Case report

We present the case of a 41 year old female patient with cTTP on prophylaxis with rADAMTS13 who underwent total hip arthroplasty for severe hip joint osteoarthritis due to Morbus Perthes.

The patient presented at our center at the age of 36 years following a first overt episode of cTTP with acute thrombocytopenia, hemolytic anemia and microthrombotic stroke during her second pregnancy. At that time, she suffered a fetal loss at 27 weeks of gestational age. Diagnostic workup revealed a severe deficiency of ADAMTS13 activity (residual activity 0.048 IU/mL, reference range 0.40–1.30, measured with fluorometric assay), with anti-ADAMTS13-antibodies (ELISA) repeatedly ruled out, confirming the diagnosis of cTTP. Genetic testing showed biallelic mutations in the ADAMTS13 gene with heterozygous missense mutation in Exon 24 and heterozygous small duplication in Exon 29 (c.[3178 C > T]; [4143dupA] I p.[(Arg1060Trp(;)Glu1382Argfs)]). Retrospectively, she may have experienced a first mild episode earlier in her first pregnancy that was interrupted for intrauterine growth retardation at a gestational week of 11, which was at that time presumed to be associated with a cytomegaly virus infection. Since the diagnosis of cTTP she was treated with prophylactic plasma infusions every 2–3 weeks, which were intensified to once weekly during the last trimester of her third pregnancy. No pregnancy complications occurred and a healthy baby was delivered at term via cesarian section. Cesarian section was covered with daily infusions of 3 units FFP for 3 days, another 2 units on day 7, followed by weekly infusions of 3 units for another 8 weeks. The patient had no signs of hemolysis or thrombocytopenia but ADAMTS13 activity was not measured. Afterwards, she continued receiving plasma infusions every two weeks because of recurrent mild hemolytic activity and cephalgia.

Because of the treatment burden of standard plasma therapy, our patient was switched to a prophylaxis with rADAMTS13 40 IE/kg body weight every two weeks in October 2024. ADAMTS13 activity under this prophylaxis was assessed at different timepoints and showed a peak level around 0.80 IU/mL, approximately 0.20 IU/mL after six days and 0.06–0.08 IU/mL after 14 days. Recombinant ADAMTS13 administration was well tolerated and effective. No other cTTP episodes occurred.

The patient underwent an elective total hip arthroplasty on the left side in February 2025. Recombinant ADAMTS13 was administered as part of her routine prophylaxis one week before surgery and to achieve sufficient ADAMTS13 activity in the perioperative setting again one day before surgery. Intraoperative blood loss was estimated at 300 mL. The patient received 1200 mL of infusion intraoperatively and 1000 mg of tranexamic acid intra-articularly. No additional infusion or red blood cell transfusion was required postoperatively. Hemoglobin concentration was 14.8 g/dL before surgery, dropped to 11.1 g/dL on postoperative day (POD) 1 and was normalized with 12.1 g/dL on POD7.

Red blood cell count was 4.82 × 10^12^/L, 3.59 × 10^12^/L and 3.89 × 10^12^/L before surgery, on POD1 and POD7, respectively.

On day 1 after surgery the ADAMTS13 activity was 0.36 IU/mL and dropped to 0.17 IU/mL on day 3 after surgery so that we decided to administer rADAMTS13 again on that day as well as 7 and 10 days after surgery due to decreasing ADAMTS13 activity levels.

Infusions of rADAMTS13 were further continued weekly until 4 weeks after surgery and then switched back to bi-weekly administrations. The platelet count remained in the normal range in the pre- and postoperative period. No thrombotic or bleeding complications occurred. The ADAMTS13 activity and the administration of rADAMTS13 in the perioperative period are shown in the Table 1.

**Table 1:** Levels of ADAMTS13 activities, platelet count and time points of rADAMTS13 administration (after trough level assessment) in the postoperative phase

Postoperative days (POD)	Administered units of rADAMTS13	ADAMTS13 activity (IU/mL)	Platelet count (10^9^/L)
−1	2500	n.a.	n.a.
1	-	0.36	171
3	2500	0.17	191
4	-	0.64	n.a.
7	2500	0.27	329
10	2500	n.a.	n.a.
17	2500	0.22	n.a.
23	2500	n.a.	n.a.
24	-	0.50	308
31	2500	0.20	n.a.
37	-	0.16	n.a.
43	2500	n.a.	n.a.
58	2500	0.06	222

*Abbreviations*: *n.a.* not available

**Fig. 1:**
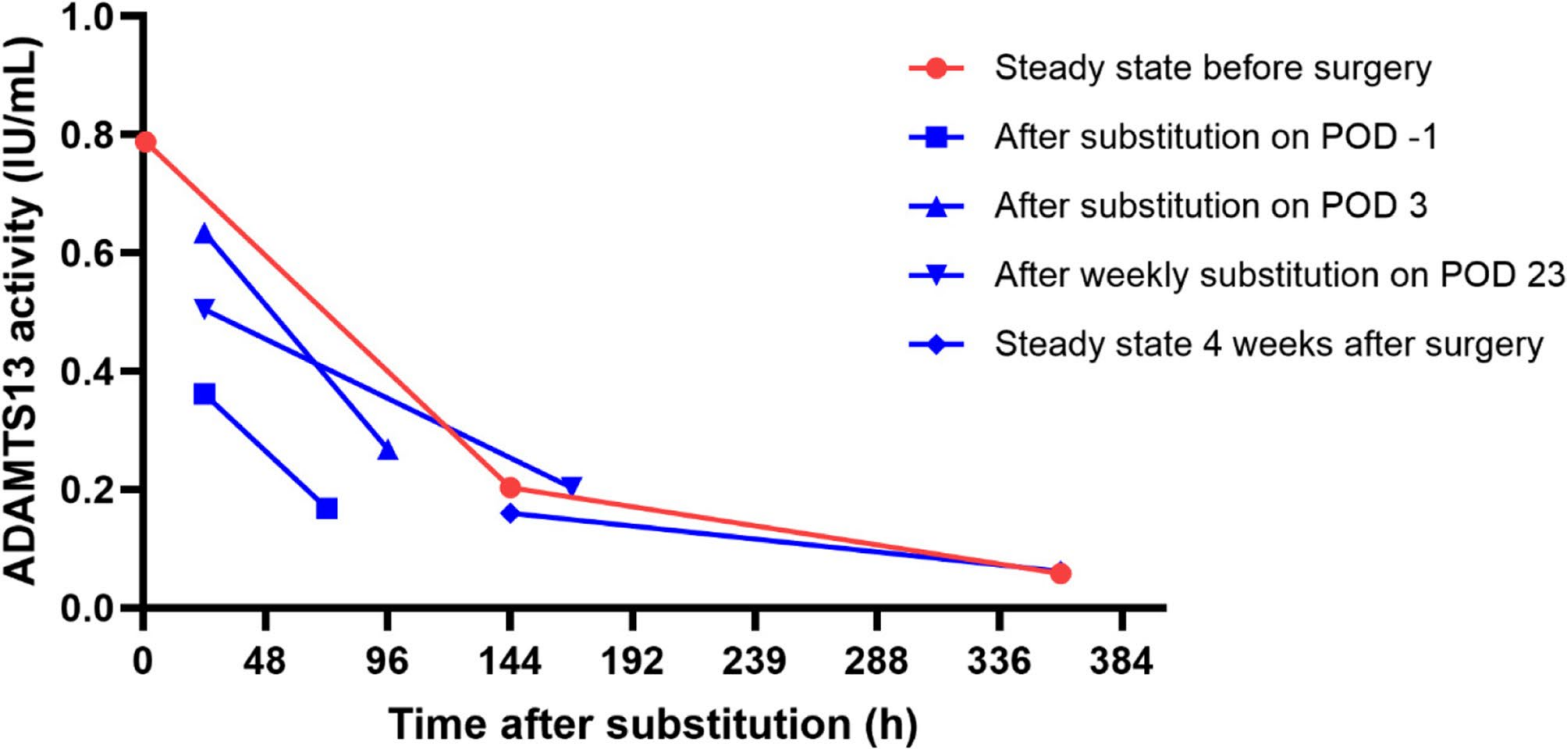
Levels of ADAMTS13 activities in steady state before surgery and during the 4 weeks after surgery

 Using a two-compartment model of distribution and elimination of ADAMTS13, we calculated an apparent half-life during the elimination phase as a surrogate parameter for the actual half-life. In the initial phase of replacement therapy with rADAMTS13, the apparent half-life in our patient was 132 h after first dosing and 119.5 h after the second dosing. We observed a marked change of these half-lives in the postoperative period: on day 1–3 post surgery the apparent half-life was reduced to 43.3 h, continually increased to 57.9 h between day 4–7 and further to 109.5 h on day 24–31 and 158 h between day 37–58 post surgery (Fig. 1). This surrogate parameter is a measure of the actual half-life of the infused rADAMTS13 and also depends on her residual ADAMTS13 activity of approximately 0.06 IU/mL. These data demonstrate that the apparent half-life of rADAMTS13 in our patient was substantially diminished in the first 4 weeks after surgery and that intensified dosing was necessary to keep ADAMTS13 trough levels > 0.2 IU/mL.

## Discussion

The ADAMTS13 activity levels during the routine prophylaxis in our patient were consistent with those reported in the phase-3-trial [7], although we initially observed even slightly higher trough levels. On the other hand, there was a more rapid decrease in ADAMTS13 activity in our patient in the postoperative phase under intensified prophylaxis with rADAMTS13 (0.64 IU/mL on POD 4 and 0.50 IU/mL on POD 24 24 h after substitution) compared to her steady-state parameters. Postoperatively, ADAMTS13 activity after 3 days was already as low (0.17 IU/mL) as after 6–7 days under steady-state conditions.

To our knowledge this is the first real-life report on the use of rADAMTS13 in elective orthopedic surgery in a patient with cTTP. Total hip arthroplasty is a hemostatic challenge that leads to an increased release of von-Willebrand-factor and decrease in ADAMTS13 activity levels also in the healthy population [8, 9]. Endothelial damage in the surgical field and therefore increase in shear stress further enhance these hemostatic alterations [3]. These mechanisms may explain the faster consumption of ADAMTS13 in our patient. We cannot exclude that hemodilution might have had an impact on lowering the ADAMTS13 activity on POD1 although the amount of blood loss and intraoperative infusion therapy was small.

In the absence of sufficient ADAMTS13 activity levels, a major surgery can provoke an acute TTP episode as reported in cardiovascular [10], gastrointestinal [3, 11] and orthopedic surgery [12–14]. These acute acquired TTP episodes typically occurred 3–9 days after the intervention. While these studies describe patients with a new onset of an autoimmune disorder, the management of an elective surgery in a patient with known cTTP is another challenge. The optimal level of pre- and postoperative ADAMTS13 activity in cTTP is not discussed in current international guidelines [15], so that the management is left up to the clinical experience of the treating physician. Arcudi et al. published a case series of seven patients with known acquired TTP undergoing elective surgery and suggested that ADAMTS13 activity levels of around 0.25 IU/mL before surgery should be achieved to safely perform the procedure [16]. Analyzing Arcudi’s and other authors data Pinheiro et al. published a small systematic review of the preoperative TTP prophylaxis in patients with acquired TTP undergoing surgery. According to this review relapse did only occur in one patient undergoing coronary artery bypass who did not receive prophylaxis and, retrospectively, showed severe ADAMTS13 activity before surgery [17]. We do believe like the mentioned data suggests that prophylaxis increasing the ADAMTS13 activity above 0.2 IU/mL is optimal to prevent relapse in patients undergoing major surgery.

Our report has some limitations. First, the assessment of antigen, activity and in particular the multimer pattern of the von Willebrand factor corresponding to the measured ADAMTS13 activity levels are lacking. This would have helped to underline the pathophysiologic hypothesis of increased ADAMTS13 consumption. Second, more data regarding the residual ADAMTS13 activity at different time points during initiation of replacement and surgical phase would be necessary to better describe the pharmacokinetics, especially rADAMTS13 initial distribution phase, in our patient.

Nevertheless, the ADAMTS13 level in our patient before surgery can be regarded as very good and sufficient. Since post-surgical inflammatory processes are associated with an increased consumption of ADAMTS13 that can continue for several days to weeks after surgery, the prophylaxis regimen should be personalized based on the clinical and laboratory parameters.

## Conclusion

The present case demonstrates that a perioperative management of cTTP with adapted dosing regimen of rADAMTS13 is safe and efficient. Individualized decisions should be made based on the clinical course and laboratory response. Further studies are needed to define the optimal ADAMTS13 peak and trough levels during the perioperative setting.

## Data Availability

Please contact author for data requests.

## Abbreviations

cTTP: Congenital thrombotic thrombocytopenic purpura
ADAMTS13: A disintegrin and metalloproteinase with thrombospondin motifs 13
rADAMTS13: recombinant ADAMTS13
FFP: Fresh frozen plasma
ELISA: Enzyme-linked Immunosorbent Assay
POD: Postoperative day
